# Current Status and Future Prospects of Clinically Exploiting Cancer-specific Metabolism—Why Is Tumor Metabolism Not More Extensively Translated into Clinical Targets and Biomarkers?

**DOI:** 10.3390/ijms20061385

**Published:** 2019-03-19

**Authors:** Magesh Muthu, Anders Nordström

**Affiliations:** Department of Molecular Biology, Umeå University, SE-90187 Umeå, Sweden; magesh.muthu@umu.se

**Keywords:** translational medicine, metabolomics, metabolism, cancer, tumor, biomarker, drug discovery, metabolic homeostasis

## Abstract

Tumor cells exhibit a specialized metabolism supporting their superior ability for rapid proliferation, migration, and apoptotic evasion. It is reasonable to assume that the specific metabolic needs of the tumor cells can offer an array of therapeutic windows as pharmacological disturbance may derail the biochemical mechanisms necessary for maintaining the tumor characteristics, while being less important for normally proliferating cells. In addition, the specialized metabolism may leave a unique metabolic signature which could be used clinically for diagnostic or prognostic purposes. Quantitative global metabolic profiling (metabolomics) has evolved over the last two decades. However, despite the technology’s present ability to measure 1000s of endogenous metabolites in various clinical or biological specimens, there are essentially no examples of metabolomics investigations being translated into actual utility in the cancer clinic. This review investigates the current efforts of using metabolomics as a tool for translation of tumor metabolism into the clinic and further seeks to outline paths for increasing the momentum of using tumor metabolism as a biomarker and drug target opportunity.

## 1. Metabolic Homeostasis and Implications for Studying Quantitative Metabolic Profiles in Tumor Biology

Homeostasis is the organism’s ability to maintain a certain steady internal condition of physiological aspects, such as blood glucose, body core temperature, or blood gases. The human body typically uses more than half of the daily energy consumption for maintaining this homeostasis through the sleeping metabolic rate [1], whereas actual physical activity accounts only for 10 to 30% [2]. These numbers are a good illustration of that cells not are in an equilibrium, in which all influences are balanced, but rather in a constant flow of energy and matter—a steady state. On a metabolic level, the homeostasis serves to deliver metabolic precursors, metabolic intermediates, and metabolic end products just in time [3] to where they are required for cellular function. The homeostasis mechanism also serves to avoid scenarios of accumulation or depletion of metabolic intermediates as this can be toxic both for individual cells and multicellular organisms. Examples of toxicity are accumulation of phenylalanine [4], 5-oxoproline [5], and lipid intermediates [6,7,8].

**Figure 1:**
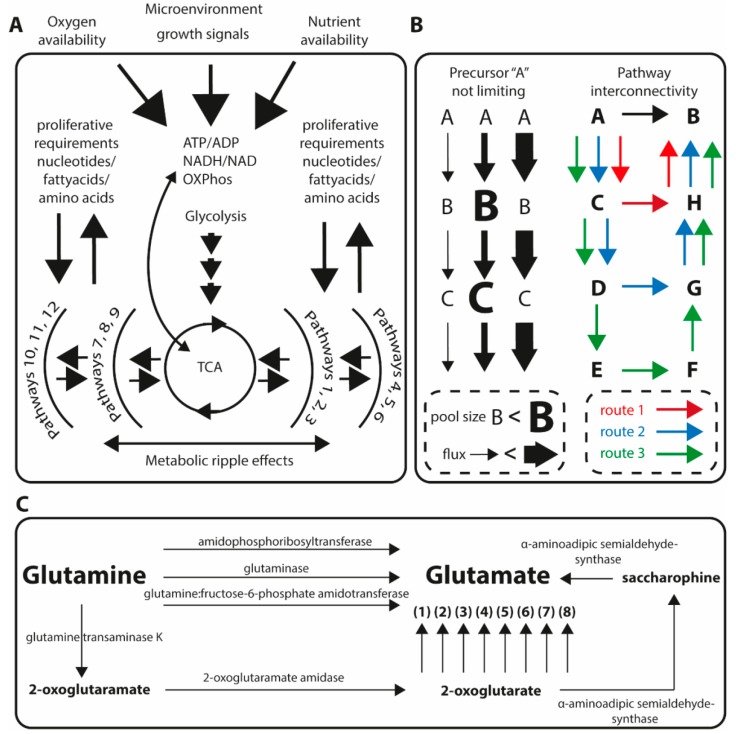
(**A**) Maintaining ATP is performed with extreme precision. This causes metabolic ripple effects throughout the metabolism. Glycolysis and tricarboxylic acid (TCA) cycle are central nodes in this. (**B**) Metabolic homeostasis can be maintained primarily by the mechanics of regulation of fluxes (left) and alternative pathways or shunts for diversion of metabolic flow. (**C**) Conversion of glutamine to glutamate is most often associated with the enzyme glutaminase (GLS). There are, however, at least 11 other paths between these metabolites with experimental evidence. (1) glutamate dehydrogenase 1 (GLUD1), (2) phosphoserine aminotransferase (PSAT1), (3) tyrosine aminotransferase (TAT), (4) aspartate aminotransferase 1 (GOT1), (5) alanine aminotransferase 1 (GPT), (6) 4-aminobutyrate aminotransferase, mitochondrial (ABAT), (7) kynurenine/alpha-aminoadipate aminotransferase (AADAT), and (8) ornithine aminotransferase (OAT).

The energy currency of the cellular machinery—ATP—is regulated with extreme homeostatic precision. Certain cells and tissues, such as skeletal and cardiac muscle, maintains ATP concentration constant even in scenarios where the demand for ATP turnover differs by two orders of magnitude [9,10]. Deviations from the ATP set point will quickly have pathological consequences, for example, chemical manipulation of ATP levels in mouse proximal tubular (MPT) cells to 25% of the ATP base level induced necrotic cell death in 50% of the cells within 12 h [11]. Conversely, exogenously added ATP has also been shown to induce dose dependent apoptosis in HL-60 and F-36P cells [12]. In fact, the intracellular ATP level has also been proposed to be a determinant of apoptotic/necrotic cell death fate [13]. On a global scale we do not have a complete picture of how the maintenance of ATP homeostasis draws and interacts with other parts of known metabolic pathway schemes. However, we do know that ATP synthesis is strongly connected with oxidative phosphorylation, which in turn depends to a large extent on the tricarboxylic acid cycle (TCA) for NADH production [14]. The TCA has several points for anaplerotic/cataplerotic reactions [15,16] and is thus a focal point for mediating the further metabolic processes required for sustaining the ATP homeostasis: for example, glycolysis (acetyl-CoA), fatty acid synthesis (citrate), fatty acid oxidation (acetyl-CoA), and glutaminolysis (α-ketoglutarate). This links ATP synthesis to the TCA, which in turn draws and interacts at multiple points in the metabolism. Thus, maintaining the ATP homeostasis will rapidly affect more distant metabolism causing metabolic ripple effects depending on nutrient status, oxygen, and microenvironment and growth signals illustrated in Figure 1a.

A hallmark of cancer cells is their unlimited ability for replication. This implies frequent cell divisions during which all material in the cell essentially doubles. Any cell division will clearly impact requirement for ATP available for protein, lipid, and RNA/DNA synthesis, and should trigger large ripple effects in the global metabolism [17]. Using synchronized cells and pulse chase stable isotope tracer experiment Shlomi and coworkers recently deciphered changes in metabolic fluxes through the TCA during cell division [18]. For example, they showed that oxidation of glucose derived flux peaked the in late G1 phase, while oxidative and reductive glutamine metabolism dominated the S phase [18].

A basic assumption is that the homeostatic mechanisms will manifest differently in cancer cells simply because their fundamentally altered metabolism that supports rapid and continuous proliferation in a competitive microenvironment where nutrient usage must be flexible [19,20] and oxygen often is limiting. At the same time a basic assumption is that the cancer cells for their survival needs to maintain their ATP homeostasis as normal cells must. Identification of specific mechanisms sustaining the global metabolic homeostasis, driven and balanced between by the cancer cells continuous proliferation and their need for maintaining ATP homeostasis, would lead to the identification of therapeutic or biomarker opportunities.

Measuring quantitative metabolic profiles with the intention to associate them with physiological or pathological conditions have been ongoing for over 50 years [21,22,23]. This was coined “metabolomics” in the literature 1998 to 2001 [24,25,26]. Gas and liquid chromatography (GC/LC) coupled with mass spectrometry is the most sensitive approach, typically with an ability to relatively compare concentration wise well over 500 annotated metabolites in a biological sample [27]. Over the last 10–15 years developments in the analytical pipeline, including high resolution benchtop mass spectrometers [28] and raw data alignment [29] software [30] and databases [31,32], to aid interpretation and data integration. Assuming that there actually is an immense difference in metabolism and metabolic homeostatic mechanisms between normal and cancer cells, it is reasonable to ask why modern day metabolomics not yet have identified additional new and specific differences to lead tumor biologists in novel metabolic avenues. There is still a lot of metabolic focus and new revival on metabolic alterations that have been known long before the arrival of metabolic profiling such as the “Warburg effect” [33], glutaminolysis [34,35,36], and the complex role of acetate both for fatty acid synthesis and oxidation [37,38]. First, one difficulty lies in the fact the homeostatic mechanisms works towards keeping concentration levels of metabolic intermediates constant avoiding toxicity [4,5,6,7,8]. Secondly, if the concentration does not differ more than ±20%, the analytical method and processing pipeline might struggle to identify the difference as significant [39]. Thirdly, from a data evaluation perspective, and ultimately from a biological perspective, a major question exists: How to value amplitude of a given difference in relation to another difference with a smaller/larger amplitude? That is, is a metabolite displaying a large fold change more important for understanding the system than a metabolite showing a smaller fold change? Consider for example oral glucose test used for probing insulin resistance, prediabetes or gestational diabetes in pregnant women. In this test 75 g of glucose in the form of a sweet juice (237 mL) is administered. After 2 h blood glucose level for a healthy individual should be below 7.8 mmol/L; between 7.8 and 11 mmol/L the person is considered to have impaired glucose tolerance and should be further diagnosed; and above 11 mmol/L the person is considered to be diabetic [40]. This means that a fold change of 1.4 makes a clear cut clinical diagnosis. In a study where the oral glucose tolerance test was administered to healthy individuals the blood glucose level varied between 5.8 and 8.1 mmol/L after two hours (1.4-fold), at the same time several fatty acids and acyl-carnitines had changed between 2- and 4-fold [41]. Diabetes is far from a fully understood disease, but if no disease mechanisms were known, would the higher blood fold change of palmitate lead us in the direction to believe that diabetes mechanistically is a disease of impaired lipid metabolism?

To decipher primary metabolic effects from secondary or tertiary is critical for understanding of mechanisms. The main cellular options to compensate or regulate accumulation or depletion of metabolic intermediates will result in secondary or tertiary effects and they are (1) to modulate activity or amount of available metabolic enzyme thus altering the metabolic flux and (2) to upregulate alternative synthetic routes, thus opening up metabolic pathway redundancy or interconnectivity. These two scenarios are illustrated in Figure 1b,c.

An increased metabolic flux through a specific set of reactions can lead to a measurable concentration increase in intermediates. But, depending on reaction kinetics, a dramatically increased flux can be completely masked in a concentration sense by matching of downstream pathway activity increase (Figure 1b). Metabolic fluxes are readily measured using stable isotope tracers [42,43], and can also, in defined pathways, be quantitatively determined [44]. However, these approaches are not as easily applied to untargeted discovery of flux alterations. Alternative biosynthetic routes are conceptually even more difficult to account for. When interpreting metabolomics data, a common approach is to map metabolites onto known metabolic pathways. There are two problems with this. (1) Metabolic pathway schemes can create the illusion of certain metabolites are far from, or close to each other spatially, when in fact our collection of two-dimensional pathway schemes are completely constructed. (2) Which reaction should a given metabolite be mapped onto? All road leads to Rome, but there are many roads to choose from. For example, using the function “metabolic route search” in the Humancyc database [31] to find metabolic routes between glutamine and glutamate, generates essentially as many routes as the inquiry limits allows. In the case glutamine to glutamate, at least 12 different routes with experimental evidence in Homo sapiens. These aspects, increased flux vs. metabolite concentrations, and the metabolic redundancy are illustrated in Figure 1b,c. Further, this also complicates multiomics overlays as each metabolite can be associated with different reactions and thus with different genes, mRNA, or proteins.

## 2. Cancer-Specific Metabolism as a Drug Target Opportunity at Present and What We Can Learn from Historical Tumor Metabolic Drug Target Discovery?

While the first FDA approved pharmacological treatment for cancer was the DNA alkylating chemical agent mustard gas [45], the second (leucovorin) and third (methotrexate) drugs approved target metabolism [45]. The importance of vitamins for normal growth was studied intensely between ~1850 and 1950, and it was discovered that many symptoms, conditions, and illnesses previously associated with infections or toxins actually was result of vitamin deficiency [46]. This led researchers to explore the role of these substances for tumor growth. The vitamin B relative, inositol (nonessential), was found to have some anti-tumor effect [47] and further studies using what was thought to be folic acid (vitamin B9), then called *L. casei* fermentation product and ultimately later shown to be pteroyltriglutamic acid, a folic acid conjugate, showed inhibiting effect on tumor growth [48,49]. Further studies using conjugates of folic acid and pteroyldiglutamic, and pteroyltriglutamic acids, in patients with terminal cancer of various kind [50] showed that cancer cells in bone marrow biopsies from patients with acute leukemia displayed accelerated growth. This made researchers hypothesize that the tumor depended more heavily on folic acid supply and that antifolate (antimetabolite) potentially could derail this dependency. Although, at this point there was no understanding of the details of folic acids role for purine biosynthesis, Sidney Farber through collaborations obtained aminopterin, and could, in a landmark 1948 paper, show that this substance induced remission in children with leukemia [51]. Aminoptrein was early on replaced by a derivative, methotrexate, and even though it was amongst the very first substances available to treat cancer pharmacologically, it is still a major player in therapeutic regimens of not only leukemia therapy, but also breast cancer [52]. In addition it has become a first-line treatment for rheumatoid arthritis [53], and is listed the World Health Organization’s (WHO) list of essential medicines. Asparaginase, which is also listed on WHO list of essential medicines, is a drug used to treat acute lymphoblastic leukemia (ALL), acute myeloid leukemia (AML), and non-Hodgkin’s lymphoma, and was discovered more or less by coincidence. Because of “…experiments undertaken for other purposes…” it was found that transplanted lymphomas in a mice model underwent complete regression when the tumor bearing mice received subcutaneous injections of normal guinea pig serum [54]. It could subsequently be demonstrated that the serum contained the enzyme asparaginase which converts asparagine to aspartic acid [55,56]. The pharmacological effect is achieved as the lymphoblastic leukemia cancer cells cannot synthesize adequate amounts of the amino acid asparagine for protein synthesis and are thus dependent on extracellular asparagine. When asparaginase is administered, the extracellular pool of asparagine is depleted, which subsequently will impact proliferation and viability of the cancer cells.

**Figure 2:**
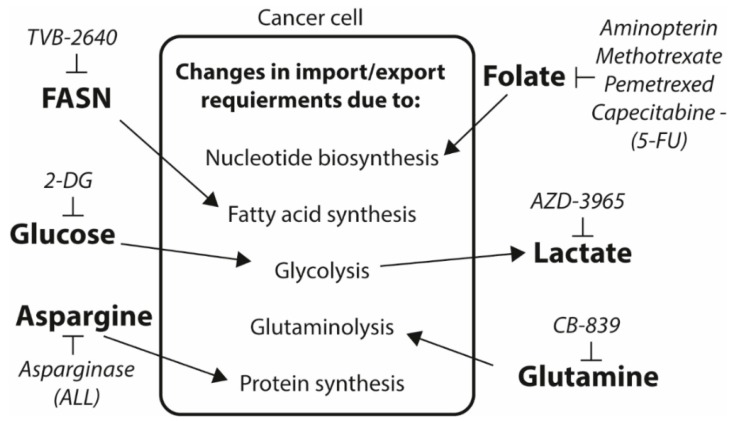
Schematic illustration of how drugs or drug targets are involved in cancer metabolic pathways.

These two examples serves to illustrate that finding basal metabolic differences in extracellular requirements (asparagine) or internal fluxes (folate) between normal cells and cancer cells is a viable route for new drug target discovery. Other well-known metabolic differences have, maybe to some surprise, not been targeted until very recently. Some of these are outlined in Figure 2. High level of glucose utilization is already exploited extensively in clinic for positron emission tomography (PET). Pharmacological direct inhibition of glycolysis has not been as successful, at least not as a single therapy. The most well studied glycolysis inhibitor is 2-deoxyglucose (2-DG). After cellular uptake of 2-DG and phosphorylation by the hexokinase, the 2-DG-P cannot be further metabolized by glucose-6-phosphate isomerase, and gets trapped and thus will 2-DG acts as a competitive inhibitor of the glycolytic pathway. However, separation of glycolysis and the pentose phosphate pathway (PPP) in yeast made cells resistant to 2-DG suggesting that blocking of glucose catabolism may not be the sole mechanism for the growth inhibitory effect of 2-DG [57]. Clinical trials using single high doses up to 300 mg/kg as adjuvant to radiation therapy for glioblastoma patients was tolerated [58,59], but for longer term adjuvant treatment, only lower doses up to 63 mg/kg is tolerated [60], which may be too low dose to achieve efficacy. The increased aerobic glycolysis in cancer cells results in increased lactate formation which needs to be removed from the cell. Inhibition of lactate transporters is therefore a rational target for disruption of cancer cell homeostasis. Phase I studies of the compound AZD-3965 is underway, with preclinical pharmacological data already reported [61]. Inhibition of glutaminolysis, which is another pathway that appears to be more critical for cancer cells, is yet another strategy for hitting tumor specific metabolism. Clinical trials using the glutaminase inhibitor CB-839 are also under way. Metabolic drug targets approved and in clinical testing are summarized in Table 1 and Table 2, respectively.

Two of the very first pharmacological substances for cancer treatment are still a mainstay of cancer therapy for several different types of cancers. These targets continue to attract research, which might further enhance their values as metabolic points of therapeutic intervention [65,66]. Interestingly, they are both targeting metabolic weak spots/weaknesses of cancer that was more or less discovered by chance. The rewired metabolism of cancer cells most likely forces them to interact biochemically differently with their surroundings which can manifest like increased dependence of extracellular asparagine or sensitivity to disruption of the folate cycle. What strategies are available today for identifying potential weak spots more systematically? Broadly, these strategies could be conceptually dived into, computational, cell-based, and in vivo approaches.

Several computational or modeling-based approaches for finding metabolic vulnerabilities have been developed. These include PRIME [67], which correlates gene expression data with a phenotypical, measurable output, such as growth and biomass for individual cell lines. When models for cancer cell are compared to those of normal cells, prediction of potential drug targets can be made. Constraint based models of metabolic networks integrates different types of mathematical and biophysical constraints, enabling prediction of metabolic phenotypes and, importantly, genes essential for sustaining growth. These models are typically applied to individual pathways [68,69]. A global reconstruction model of human metabolism is publically available—Recon 2 [70]—this model can be combined with public clinical and experimental gene transcriptome data in order to identify potential metabolic characteristics and thus weaknesses of specific cancer types [71].

Both methotrexate and asparaginase were based on discoveries made directly on human biopsies and in vivo studies after intervention. However, systematic studies imply large biochemical spaces to be investigated. For reasons of cost and throughput, cellular models are preferred, even if we know that these are potentially poor representations of in vivo conditions. There are however several recent examples of development that could advance cellular models systems for cancer drug target discovery. Modification of growth media to better represent human plasma resulted discovery of de novo pyrimidine biosynthesis inhibition [72]. This could be traced to an inhibitory effect of uric acid on UMP synthase. Interestingly, uric acid has ~10-fold higher concentration in humans compared to mice. Stable isotope tracing and metabolomics assisted in deciphering that the mitochondrial glutamate transporter is crucial in KRAS mutation colon cancer cells ability to sustain aspartate derived amino acid and polyamine synthesis [73]. Evaluating effect of nutrient availability over prolonged periods of times can be studied using a Nutrostat for competitive proliferation assays across multiple cell lines [74]. Development of 3-D culturing methods to better represent epithelial cell growth through a cell sheet system was recently employed to screen sensitivity towards chemotherapeutics [75]. Models like this could be useful for systematic investigation of cancer cell communication with extracellular matrix to identify dependencies that are essential for the cancer cells. Interestingly, from a metabolomics perspective, the scaling factor of the cell system under investigation does not appear to be limiting for the number of metabolites possible to measure in cell lysates [76].

Studying tumor growth for the identification of metabolic limiting factors in vivo poses the largest challenge both technically and in terms of experimental design possible to achieve for cost and ethical reasons. There are however a growing number of reports where metabolic aspects of cancer progression has been examined in animal models. Plasma metabolome changes were followed over time during breast tumor growth and metastasis in mice revealing changes in arginine metabolism as well as changes in structural and signaling lipids that correlated with metastatic phase [77]. Metabolomics measurement of tumors with different inclination for metastasis formation unveiled sialic metabolism as upregulated in highly metastatic tumors [78]. Genetic knockdown inhibiting biosynthesis of activated sialic acid resulted in less metastasis forming in vivo [78]. Pharmacological inhibition of mTORC can suppress cancer cell proliferation because of its role in controlling glycolysis. Direct perfusion with stable isotope-labeled glutamine into mouse tumor and measurement of glutamine derived aspartate as well as tumor glutamine uptake suggested glutaminolysis mechanisms compensating for suppressed glycolysis, which in turn could be restricted using an inhibitor of glutaminase [79]. An in vivo screening approach using patient derived tumor cells in to which lentiviral shRNA targeting 12 different surface transporters were introduced [80]. These cells were pooled and introduced into mice with tumors being harvested at different time points. By measuring the frequency of these shRNA over time it was deduced which ones that prevented tumor growth and, carbonic anhydrase IX could be identified together with others as essential for tumor formation [80]

## 3. Does Cancer Cell Metabolism Leave a Disease Specific Metabolic Imprint That Can Be Used as a Biomarker?

Arguably, biomarker discovery, is the area of altered cancer metabolism that has attracted most research with >1500 publications returned through https://pubmed.gov search on “cancer” and “metabolomics” and “biomarker”. Most of these are published since 2008. Finding biomarkers is not only interesting from a diagnostic perspective. Biomarkers often arise from a pathology specific biochemistry, and may thus lead to new drug targets as well. There is a clear rationale for assuming that tumor elements can reach the blood stream through for example mechanisms of direct export, necrosis, apoptosis, microvesicle budding, or phagocytosis. It is therefore conceivable that direct biochemical evidence of tumor presence is possible to measure in the blood. With an altered metabolism in cancer cells, there is a possibility that this altered metabolism will be reflected in the blood and potentially used as biomarker. However, there is general problem with metabolic biomarkers, the “metabolites” are generic, and that is there is no tumor unique metabolite. All metabolites are potentially present in all cells as energy, signaling, or structural elements. Also, the altered tumor metabolism shifts the fluxes through individual metabolite pools and potentially skews the relative proportion of specific metabolite pairs. This is further complicated by the following. (1) Since the tumor in most cases is very small in relation to the rest of the body, there is a fair chance that a tumor specific metabolic profile leaking out from tumor cells is diluted and disappears in the blood volume where a large contribution to the blood metabolic profile comes from metabolic active muscle cells which constitute approximately 40% of human body weight. (2) The tumor triggers innate immune responses such as inflammatory responses, which, in turn, create much larger alterations to the blood metabolic profile than the primary changes leaked out from the tumor cells. This has been observed when comparing the blood metabolic profiles of nonrelated diseases where a majority of metabolic differences were found not to be disease specific [81]. (3) The blood metabolic profile will reflect what we eat and our life style which further convolutes the cancer-specific metabolites leaking out from the tumor. Because of (1), (2), and (3), blood metabolic biomarkers can be more complicated to assign than for example cancer-specific DNA methylations which can achieves specificities in clinical cohorts close to 100% [82]. In fact, a recent study where a multi panel of levels of certain proteins and mutations of free circulating DNA was applied to different tumors in different organs, reported not only 99% specificity but also showed that it was possible to assign the organ in which the tumor was localized for 83% of the patients [83]. This has raised the question of the potential of universal cancer screening, as opposed to organ based cancer screening for diagnostic purposes [84].

Despite the massive amount of metabolomics literature reports proposing tentative biomarkers for various cancers, no single metabolite or metabolite panel have found clinical utility over the last 10 years. A recent review compiled various biomarkers, with some sort of validation for several different pathologies including cancer, obtained through metabolomics [85]. Many of these validations included running comparable cohorts at a separate point in time, i.e., comparing cancer cohort 1 with healthy control cohort 1 and, subsequently, validating metabolic differences by comparing cancer cohort 2 with healthy control cohort 2. One possible reason to that none of these “validated” biomarkers have reached to the clinic yet is that there is no mechanistic explanation for their presence and this lack of biochemical understanding makes the “clinic” reluctant to perform further costly validations, clinical trials and implementation. If the number of studies or “validations” supporting an association between a biomarker and a specific type of cancer was then sole driver for clinical implementation, metabolites frequently associated with that cancer should attract clinical interest. We tested this hypothesis by querying (https://pubmed.gov) using the words “breast cancer”, “biomarker”, and “metabolomics”. We found 69 studies using metabolomics that were associating annotated metabolites with breast cancer in some sense in either clinical patient material or cellular models. We subsequently grouped the resulting table by the number of reports a specific metabolite is associated with breast cancer. The result for all metabolites reported at least twice is shown in Table 3. The amino acid glutamate comes out at the top with 13 reports. Is glutamate a good biomarker candidate for diagnosis of breast cancer? Most likely not. Altered biofluid glutamate levels is also associated with seizures in Wilson’s disease [86], cardiopulmonary bypass in infants [87], probable Alzheimer’s disease and depression [88], the relationship between lung cancer and chronic obstructive pulmonary disease [89], and multiple sclerosis [90], to mention just a very small subset of reports on glutamate association with various pathology. Mechanistically, glutamate could be associated with cancers in terms of increased glutaminolysis (cancer cells dependency of extracellular glutamine) which through the action of glutaminase enzyme, converting glutamine to glutamate (Figure 1c). But, since glutamate clearly is associated with many other pathologies, the observations of altered glutamate levels in cancer patients is most likely mechanistically not directly linked to the cancer itself, but rather to secondary biochemical events such as inflammatory responses and thus not interesting to pursuit for further clinical validation of its role in the cancer context.

Based on Table 3, it seems as if a significant number of deregulated metabolites in cancer samples were reported extensively, yet none have translated to the clinic and assuming their biochemical specificity towards tumor development is most likely an oversimplification for several reasons: (i) flawed understanding in differentiating tumor versus normal metabolites/metabolite levels and (ii) unavailability of multidisciplinary approach to understand the cancer metabolism that can directly or indirectly relate to the root of the pathological gene regulation.

## 4. Future Prospects for Exploiting the Altered Cancer Metabolism as Drug Target and Biomarker

The altered cancer metabolism offers obvious drug targets and is a potential source of biomarkers. It is a bit strange that cancer cells addiction to glutamine (known since 1956) and necessity for exporting lactate (known since Warburg’s discovery 1923) has not been subject to clinical trials until 2018 with CB- 839 and AZD-3965, respectively. Clearly, there is still a lot of drug discovery to be made in the well-known areas of unique cancer metabolism.

Life cannot sustain too large deviations from an ideal metabolism. Homeostatic mechanisms maintain this ideal metabolism during cell division and under conditions of changes in nutrient and oxygen supply. The differences that we can expect to find when comparing cancer cells and normal cells is how this homeostasis is maintained through altered fluxes and alternative metabolic routes. Identifying differences further away from central aspects such as aerobic glycolysis and glutamine addiction may provide better therapeutic windows. The homeostatic mechanisms complicate discovery as quantitative differences may not be apparent and, to address this, global flux probing methods are being developed [42,43,150,151,152]. From a basic understanding of cancer cell nutrient requirements, it could be worth revisiting classical experiments [34,36] investigating cellular needs with more powerful metabolomics and flux techniques available today.

The quest for metabolic biomarkers of cancer has been intense over the last decade. One possible explanation to the lack of metabolic biomarker panels being evaluated in clinical trials so far is that many of the proposed biomarkers lack clear and proven mechanistic link to the cancer metabolism. Identification of metabolic blood biomarkers directly in patient samples is apparently difficult. This can be rationalized by considering that tumors are small relative to the rest of the body and that a unique cancer metabolic profile leaking out into the blood easily can get masked by the same metabolites leaking out from normal cells of slightly different proportions. The unique tumor profile will become diluted in the blood. It is also conceivable that innate immune responses further masks tumor specificity. However, if we look how metabolic biomarkers for other diseases were developed, it appears as if the biomarker and its detection were developed from known pathological biochemistry. For example, the discovery of *Helicobacter pylori* expressing urease [153] and the subsequent exploitation of urease activity for developing a stable isotope labeled urea test and for detecting the labeled CO_2_ arising from the urease activity [154]. With the powerful analytical technology available today, cancer biologists should join with analytical chemists to explore strategies for employing well characterized cancer biochemistry for diagnosis. It would be unfortunate to have a scenario like for phenylketonuria (PKU) diagnosis for which 30 years passed between discovery of the pathological biochemistry and a reliable diagnostic test [155,156].

## Figures and Tables

**Table 1:** Approved drugs targeting specific metabolic aspects of the cancer metabolism.

Target	Drug	FDA Status in Cancer
Dihydrofolate reductase (DHFR)	Methotrexate Pemetrexed	Approved
5-phosphoribosyl-1-pyrophosphatase (PRPP) amidotransferase	6-mercaptopurine (6-MP) 6-thioguanine (6-TG)	Approved
Thymidylate synthase (TS)	5-fluorouracil (5-FU) Capecitabine	Approved
DNA polymerase/ribonucleotide reductase (RnR)	Gemcitabine Cytarabine	Approved
Circulating asparagine	L-Asparaginase	Approved
mutant Isocitrate dehydrogenase 2 (IDH2)	Enasidenib (AG-221)	Approved

**Table 2:** Drugs targeting cancer metabolism currently in clinical trials.

Target	Drug	Clinical Status	Cancer Type
Dihydroorotate dehydrogenase (DHODH)	Leflunomide	Phase II/III	Prostate Cancer
S6K, 4E-BP1, AMPK	Metformin	Phase II	Breast Cancer
Mutant Isocitrate dehydrogenase 2 (IDH2)	AG-120 IDH305 AG-881	Phase I/II	Hematologic malignancies and Solid tumors
Glutaminase 1	CB-839	Phase I/II	Renal Cell Carcinoma (RCC), Melanoma, Non-Small Cell Lung Cancer (NSCLC)
Monocarboxylate transporter 1 (MCT1)	AZD3965	Phase I/II	Advanced Solid Tumors, Diffuse large B cell lymphoma and Burkitt’s Lymphoma
Fatty Acid Synthase (FASN)	TVB-2640	Phase II	Advanced HER2 positive Breast Cancer, Colon Cancer, Astrocytoma
Arginine Deiminase	ADI-PEG 20	Phase I/II/III	Hepatocellular Carcinoma [62], Acute myeloid leukemia (AML) [63], advanced pancreatic adenocarcinoma [64]
α-Ketoglutarate dehydrogenase complex	CPI-613	Phase II	Advanced and/or metastatic solid tumors

**Table 3:** Query https://pubmed.gov using keywords “metabolomics”, “breast cancer”, and “biomarker”. Sorting metabolites reported by frequency.

Metabolites (Deregulated)	Number of Reports	Sample Type	References
Glutamate	13	Plasma, Serum, Tissue, Cells	[91,92,93,94,95,96,97,98,99,100,101,102,103]
Lactate	12	Plasma, Serum, Tissue, Cells	[91,94,98,104,105,106,107,108,109,110,111]
Phosphatidylcholines (PCs)	12	Saliva, Blood, Plasma, Tissue, Cells	[97,105,108,112,113,114,115,116,117,118,119,120]
Alanine	9	Plasma, Serum, Blood, Tissue, Pleural and Peritoneal effusions, Cells	[94,95,97,103,106,121,122,123,124]
Taurine	9	Saliva, Serum, Plasma, Tissue, Cells	[93,95,97,105,108,112,125,126,127]
Phenylalanine	7	Serum, Urine	[94,96,98,101,128,129,130]
Histidine	7	Plasma, Serum, Whole blood	[91,96,98,103,106,130,131]
Choline	6	Serum, Tissue, Cells	[91,94,97,108,118,132]
Lysophosphatidylcholine (LPCs)	6	Saliva, Serum, Plasma	[117,120,122,133,134,135]
Creatine	6	Serum, Urine, Tissue, Cells	[94,97,108,109,118,136]
Glucose	6	Serum, Plasma, Tissue, Pleural and Peritoneal effusions	[100,101,112,124,129,131]
Glycerophosphocholine (GPC)	6	Serum, Ductal fluid, Tissue, Cells	[108,116,118,133,137,138]
Proline	6	Serum, Plasma, Cells	[91,94,103,122,129,136]
Glycine	5	Serum, Tissue	[94,98,104,108,112]
Threonine	5	Serum, Plasma, Cells	[97,102,106,130,139]
Tyrosine	5	Serum, Urine, Cells	[91,94,98,128,136]
Isoleucine	4	Serum, Cells	[94,98,106,139]
Lysine	4	Saliva, Serum, Plasma, Blood, Tissue	[100,123,126,129]
Tryptophan	4	Serum, Plasma, Urine	[102,128,133,140]
Acetate	3	Serum, Urine, Cells	[94,97,109]
Arginine	3	Serum, Plasma, Cells	[77,141,142]
Caproic acid	3	Blood, Plasma, Urine	[125,143,144]
Glutamine	3	Serum, Cells	[97,136,139]
Guanidinoacetate	3	Tissue, Cells	[97,105,145]
Sarcosine	3	Plasma, Tissue, Cells	[103,136,146]
Succinate	3	Tissue, Urine	[109,121,147]
Valine	3	Plasma, Cells	[98,106,122]
β-hydroxybutyrate	2	Serum	[91,94]
Aspartate	2	Serum, Plasma	[95,148]
Carnitine	2	Ductal fluid, Cells	[136,137]
Citrate	2	Serum, Cells	[94,138]
Cysteine	2	Serum, Cells	[133,136]
Formate	2	Serum	[91,94]
Leucine	2	Serum	[94,98]
Myo-inositol	2	Tissue, Cells	[97,108]
Pyruvate	2	Serum, Pleural and Peritoneal effusions	[96,124]
Serine	2	Serum, Cells	[133,136]
Spermidine	2	Serum, Cells	[140,149]
